# A Facile Way to Prolong Service Life of Double Base Propellant

**DOI:** 10.3390/ma11112236

**Published:** 2018-11-10

**Authors:** Shixiong Sun, Song Ma, Benbo Zhao, Guangpu Zhang, Yunjun Luo

**Affiliations:** 1 School of Materials Science and Engineering, Beijing Institute of Technology, Beijing 100081, China; sunshixiong1989@126.com (S.S.); ms2234056@163.com (S.M.); zhaobenbo@163.com (B.Z.); guangpu_0507@126.com (G.Z.); 2 Key Laboratory for Ministry of Education of High Energy Density Materials, Beijing 100081, China

**Keywords:** service life, mechanical properties, NG migration, double base propellant, polytetrafluoroethylene

## Abstract

The safe storage time for double base propellant (DBP or DB propellant) with stabilizers could usually be calculated to be greater than 40 years. However, the actual service life is far below that, which is largely caused by the decline of propellant mechanical performance. In this work polytetrafluoroethylene (PTFE) was introduced into the double base propellant formula as an additive. The tensile properties of this propellant before and after artificial aging were determined. The evaporation and diffusion characteristics of nitroglycerin (NG) in propellant were evaluated by thermogravimetry analysis (TGA). The results showed that mechanical properties of propellant were improved due to PTFE, especially for elongation at −40 °C, which was greatly increased by 115%. Moreover, the results of TGA showed that NG migration was reduced due to PTFE, which delayed the decline of propellant mechanical performance during aging. The reduction in elongation at −40 °C caused by aging was decreased by 68.5% for PTFE modified DBP. Enhanced mechanical properties and reduced NG migration could potentially prolong propellant service life.

## 1. Introduction

Double base (DB) propellant has been widely applied to solid rocket motors due to its various advantages, such as being smokeless, having abundant raw material sources, and so on [1,2]. Nitrocellulose (NC) and nitroglycerin (NG) are the two basic components of DB propellants. For meeting the practical demands in rocket motors, DB propellants with different performances are produced by varying the mass ratio of NC/NG. However, those nitrate esters are intrinsic instable. To suppress the decomposition and prolong the safety storage time, a series of stabilizers have been developed. Zayed et at. [3,4,5] investigated the stability of DB propellants with different stabilizers using thermal analysis. Trache et al. [6,7,8] studied the impact of aging on chemical and mechanical properties of DB propellants with diphenylcarbamide stabilizer and made a good summarization on the stabilizers. Bohn et at. [9,10,11,12,13] conducted a lot of work on stabilizer depletion and prediction of propellant life. Zhao et al. [14] investigated the depletion of stabilizer in 81 kinds propellants using artificial accelerated aging test. Still there are many other meaningful works [15,16,17,18,19,20] that focus on stabilizers of nitrate esters. All the works mentioned above demonstrated that many kinds of stabilizers could significantly delay the decomposition of energetic nitrate esters. From this point of view, safe storage life of DB propellant with stabilizers could often be predicted as long as ca. 40 years [14,21]. However, the practical safe use time for almost all the propellants is generally shorter than predicted lifetime. Many factors may contribute to this phenomenon, among which the decline of propellant mechanical performance bears the primary responsibility. It is well known that NG content is of vital importance for propellant mechanical, thermal, and ballistic properties [2,22]. However, NG migrates readily in NC matrix as small molecules. Thereby, the evaporation and migration of NG in DB propellant are considered a subject of great importance [22,23,24]. Its improvement is always a focus for researchers via a simple method.

To prevent NG content reduction, polymers, such as unsaturated polyester, polyurethane, silicone rubber, epoxy resin, and so on, with excellent migration resistance ability were often chosen as inhibitions [24,25]. Many studies focus on inhibitions have been conducted [26,27,28]. They showed that appropriate inhibitions could prevent NG migration to a large extent. However, evaporation of NG is sometimes inevitable. In this regard, much less attention has been paid on NG evaporation. Since evaporation readily is intrinsic property of NG molecule, using other energetic plasticizers to dissolve NC may obtain desired volatility. For this reason, we have introduced N-butyl-N-nitratoethyl nitramine (Bu-NENA) to DB propellant and studied its evaporation characteristics [29]. It showed much lower volatilization rate for Bu-NENA/NC propellant than that of NG/NC propellant as we expected. However, the energy level of Bu-NENA is much lower than that of NG, which decreases the energy level of DB propellant. Some other plasticizers [30,31,32], such as diethyleneglycol dinitrate (DEGDN), TEGDN (TEGDN) and trimethylolethane trinitrate (TMETN) could also dissolve NC [1]. However, their energetic levels are usually less than that of NG as well. In addition, these methods cannot improve the brittleness of propellant significantly. Thereby, a facile way, which could delay NG mass loss and promote mechanical properties for DB propellants, is still desired.

It has been proved that PTFE could enhance mechanical properties of composites via a network structure [33]. In view of the crystallinity of PTFE, the abundant PTFE fibers may act as inhibitions in a sense dispersed in propellant matrix, reducing NG mass loss during aging. As mentioned above, PTFE was simply mixed into double base blends in this work. Mechanical properties of before and after artificial aged DB propellant were determined and the evaporation and diffusion of NG were studied. It shows that diffusion and evaporation of NG were limited to some extent and their mechanical properties were promoted significantly by introducing PTFE. These improvements provide a simple way potentially prolonging propellant actual service lifetime.

## 2. Experimental 

### 2.1. Materials

NC (12.0% N), nitroglycerin (NG), and dimethyldiphenylurea (C_2_) were obtained as a mixture named double base absorbed drug (the raw material of NC/NG mixture from factory with the components of NC/NG/C_2_ 51.5/47.5/1 mass %) from Shanxi Northern Xing’an Chemical Industry Co., Ltd., China. C_2_ is a stabilizer for NC and NG. PTFE (25 μm, from supplier) were obtained from Ji’nan Jinhui Chemical Co., Ltd., Ji’nan, China.

### 2.2. Propellant Preparation

**Figure 1:**
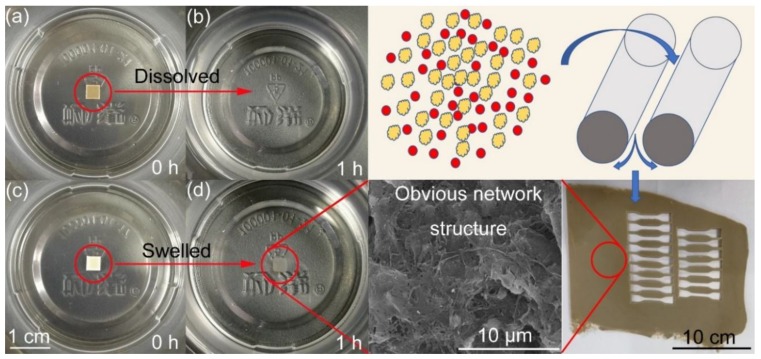
Digital images (**left**) and SEM images (**right**) of propellant dissolved in acetone. (**a**) DB propellant, 0 h; (**b**) DB propellant, 1 h; (**c**) PTFE-DB propellant, 0 h; and (**d**) PTFE-DB propellant, 1 h.

DB propellant with 6 wt% PTFE (PTFE-DB propellant) ca. 2 mm and 0.4 mm thick were prepared through a solvent-free method. The PTFE was additionally added into the propellant composition, thus, it does not change the mass ratio of the double base absorbed drug. DB propellant without PTFE was also prepared as a control. The propellants were artificially aged at 95 °C under ambient conditions. A simple scheme of propellant preparation is shown in Figure 1.

### 2.3. Characterization and Analysis

#### 2.3.1. Mechanical Properties Test 

Propellant tensile properties were conducted on the AGS-J Electronic Universal Testing Machine (Shimadzu Corporation, Kyoto, Japan) with China Military Standard GJB770B-2005 413.1. The conditions were: temperature −40 °C, 20 °C, and 50 °C; tensile rate 10 mm/min.

#### 2.3.2. SEM Measurement

To determine the distribution of PTFE in the double base propellant matrix, a piece of PTFE-DB propellant with its size 4 mm × 4 mm × 2 mm was dissolved in acetone for 1 h. Then it was filtered and vacuum dried for 2 h at 50 °C. The sample of blank DB propellant was also prepared as a control. The morphology characteristics of the two propellants were obtained on a cold field scanning electron microscopy scanning electron microscopy (SEM) (Hitachi, S4800, Tokyo, Japan). An ultrathin conductive coating was deposited before it was analyzed. 

#### 2.3.3. Thermal Property

The non-isothermal property of DB propellant and PTFE-DB propellant was investigated by thermal gravimetric analyzer (TGA) (METTLER TOLEDO, TGA STARe System, Zurich, Switzerland) at the heating rate of 10 K/min from 30 °C to 600 °C. The isothermal thermogravimetry of the thin plate samples were heated at 50–100 °C with increment of 10 °C to investigate the kinetics of NG evaporation. Both the isothermal thermogravimetry and non-isothermal decomposition of samples were tested under nitrogen atmosphere with a flow rate of 40 mL·min^−1^.

#### 2.3.4. Crosslinking Density

The crosslinking density of DB propellant and PTFE-DB propellant before and after aged were investigated by a VTMR nuclear magnetic resonance crosslinking density analyzer (Shanghai Niumag Electronic Technology Co., Ltd., Shanghai, China) at 90 °C.

## 3. Simulations

We performed molecular dynamics (MD) simulations to study the diffusion of NG in NC/NG and NC/NG/PTFE systems using Forcite code in Material Studio 7.0 (Accelrys Inc., San Diego, CA, USA). To be specific, NC (${\bar{X}}_{n}$ = 20), NG and PTFE (${\bar{X}}_{n}$ = 12) molecules were separately constructed and relaxed firstly using COMPASS forcefield. Then two amorphous cells (50 Å × 50 Å × 50 Å) with periodic boundary conditions were built. One was filled with NC/NG, the other one was filled with NC/NG/PTFE. The PTFE is added to the mixture additional, thus, the mass ratio of NC/NG in the latter cell are the same as that in the former one. It is worth noting that we replaced C_2_ by NC/NG 51.5/47.5 mass % (the same as the mass ratio in NC/NG raw material) in simulated samples to simplify the cell. Thereby, the mass ratio of NC/NG in simulated samples is 52.0/48.0 mass %, which exclude C_2_ mass % corresponding to experimental samples. It is similar for case in PTFE-DB propellant. Based on the fact that the content of C_2_ is the same in the two propellant and its content is very low (1 wt%) when comparison with that of NC and NG, we believe that this difference in concentration could not bring in obvious influence on the final results. 

The densities of these cells containing NC/NG and NC/NG/PTFE were 1.56 g·cm^−3^ and 1.61 g·cm^−3^ (practical case), respectively. The time step was 0.5 fs in all calculations. The computational accuracy of a “Fine” level was used in which the r_c_ is 15.5 Å. The Verlet algorithm was used to integrate equations of motion. Atom based method was used to study the coulombic interactions and van der Vaals interactions in the two cells. The Nosé-Hoover thermostat [34,35] was used to maintain constant temperature conditions. The temperature was chosen at 50–120 °C with increment of 10 °C which is the same as the temperature for isothermal thermogravimetry analysis. The pressure was chosen at one atmosphere.

The total simulation time is 800 ps. During simulation, a 350 ps NVT dynamic process were performed to overcome energy barrier and to relax all the structures, after which the systems were at equilibrium (energy profiles came to a stable value). Then we executed a 200 ps NVT dynamic process to obtain the mean square displacement (MSD~t) curves of NG in two cells from which we calculated the diffusivity of NG. A typical energy profiles in dynamic simulations and the corresponding MSD vs. t curve are shown in Figure S1 and Figure S2 (in Supplementary Materials), respectively. 

## 4. Results and Discussion

### 4.1. Morphology of PTFE in Propellant

The SEM images of PTFE-DB propellant after dissolving treatment is shown in Figure 1. It is worth noting that blank DB propellant dissolved in acetone completely, while PTFE-DB propellant maintained its shape with a fraction of double base components close to surface dissolved. It is obvious that an “entangled network” runs through the propellant. In view of the excellent mechanical performance of PTFE, the propellant mechanical properties may be enhanced. Since the NG molecules cannot enter or pass the crystal PTFE readily, the abundant PTFE fibers may act as inhibitions in a sense and suppress the diffusion and evaporation of NG.

### 4.2. Tensile Properties of DB Propellants

Table 1 shows the tensile performances of blank DB propellant (A) and PTFE-DB propellant (P6). The mechanical properties of the two artificial aged propellants were also determined as shown in Table 1. The typical stress-strain curves of the two propellants can be found in Figure S3 in supporting information. It is well known that DB propellant is brittle at low temperature due to the frozen rigid NC molecules. This is a fatal drawback of DB propellant restricting its application field. Generally, elongation of DB propellants at −40 °C largely depends on the mass ratio of NC/NG [1,2]. In this paper, elongation at maximum strength of propellant A is only 6.34% at −40 °C. This is mainly due to the relatively lower NG concentration. However, it is quite different for PTFE-DB propellant. The elongation of PTFE-DB propellant was enhanced by 115% to 13.6%. In addition, the tensile strength was increased by 71.0% from 3.28 MPa to 5.61 MPa. Both strength and elongation were improved obviously. This is of practical importance for potential broadening the application field of DB propellant. PTFE deformed to “network structure” during process of propellant (see in Figure 1). Thus, this promotion of propellant mechanical performance is mainly caused by the PTFE network structure since many works [33,36] have shown improved strength due to this kind of network.

**Figure 2:**
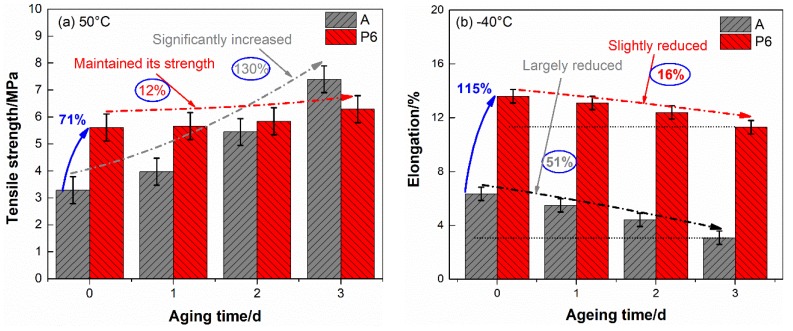
Tensile strength (**a**) and elongation at maximum tensile strength (**b**) of aged propellants.

After aged, the tensile properties of the two propellants reduced to some extent. To achieve the trend of these alteration clearly, the most concerned tensile strength at 50 °C and elongation at −40 °C were drawn in Figure 2 as a function of aging time. 

From Table 1 and Figure 2 we can see that the mechanical properties of blank DB propellant are sensitive to aging time. The tensile strength and elongation present opposite trend along with aging of propellant. The tensile strength at 50 °C increased by 130% after aged three days, accompanied by a 43.1% reduction in the elongation. In addition, the elongation at −40 °C decreased by 51.4% to a very low level (3.08%). In view of the complex loadings in handling, transportation and launch mission, such a low elongation leads to structural failure readily, which may result in deadly accident in rocket motors. This is largely responsible for much decreased actual service lifetime than theoretical predicted safe storage life for DB propellant grain. 

As for PTFE-DB propellant, the tensile strength at 50 °C and elongation at −40 °C after aged three days are 6.29 MPa and 11.4%, respectively. This means that the tensile strength maintained its level even after aged three days. Though the elongation at −40 °C slightly decreased by 16.2%, it is still at a high level which is even significantly superior than unaged DB propellant. 

Some principal mechanisms [7] may involve in the chemical changes that associated with mechanical performance of DB propellant along with aging. (1) Cross-linking hardening occurred and a three-dimensional (3D) structure was developed. During aging the decomposition of NC and NG could occur since they are intrinsic instable. Then many kinds of radicals were produced and they reacted in a complicated way [8]. Chain termination reaction may occur between alkoxyl radicals or the radicals of its ramification in this process, resulting in cross-linking between NC binder molecules. The followed elimination of free volume and inhibition of segmental rotation could introduce an increase in both modulus and T_g_. (2) Concentration profile of nitroglycerine changed. This may be due to the diffusion of NG from propellant center to its surface, as well as NG evaporation and decomposition. This reduction could also result in increasing in modulus and T_g_; (3) Break of NC chain resulted in a reduction in its molecular weight. The depolymerization process could lead to decreasing in modulus and T_g_ due to the so called “self-plasticization” effects. Both (1) and (2) would result in the increase of tensile strength and reduction of elongation, while (3) would lead to opposite results. In view of the aged samples in this paper are relatively thin (2 mm), the decline of NG content may be the key factor contribute to the changes of propellant mechanical properties. 

To verify this view, low field nuclear magnetic resonance was used to determine the crosslinking density of DB propellant as shown in Figure S4. No obvious alternation (very slightly increased) of crosslinking density can be found for DB and PTFE-DB propellant. Thus, rare 3D structures were formed during aging. It proves that crosslinking is not a main factor leading to obvious decline of propellant mechanical properties. In addition, the molecular weight between crosslinking points varies little. This infers that there is no obvious break of NC chains as well. Thus, no obvious reduction in tensile strength was obtained due to depolymerization of binder.

Besides, the reduction of NG content was determined via a mass loss way. The results are shown in Table S1 in supporting information. It can be found that the NG concentration reduced by 25.6% after aged 3 days for DB propellant. That is to say large amount of NG lost through evaporation. While it decreased by 14.0% for NG in PTFE-DB propellant. Thereby the NG content obvious differs between these two aged propellants, which would lead to different trend of mechanical properties to aging time. It is worth noting that a 14.0% reduction could also lead to obvious decline of propellant mechanical properties. However, the greatly enhanced performance is largely depending on the strong network structure. And it may be free of the influence caused by NG reduction since PTFE is stable to most of solvents. Thus, the mechanical performances were maintained for PTFE-DB propellant.

Based on mentioned above, we can conclude that (1) little three-dimensional structure was developed and little break of NC chain occurred in a relatively short aging time for both DB and PTFE-DB propellant. (2) a great deal of NG volatilized for the DB propellant sample with thick of 2 mm. Thereby, the alteration of mechanical properties of DB propellant depends on the reduction in NG content during aging. Largely reduced plasticizer decreased the free volume and inhibited the movement of NC chain segment. Thus, the tensile strength at 50 °C increased while the elongation decreased. (3) NG loss was largely reduced for PTFE-DB propellant. The strong network structure and decreased loss of plasticizer delayed the decline of propellant properties.

In a word, the mechanical properties and its stability is largely promoted due to PTFE. The improved performance may reduce the possibility of structural failure, which, as a consequence, is likely to prolong the practical application lifetime. 

Though the conclusion that PTFE-DB propellant has a relatively lower volatilization is drawn, the molecular motion characteristics of NG in DB matrix is unknown. To investigate the influence of PTFE on NG diffusivity, thermal analysis was carried out as follows.

### 4.3. Thermal Properties of DB Propellants

**Figure 3:**
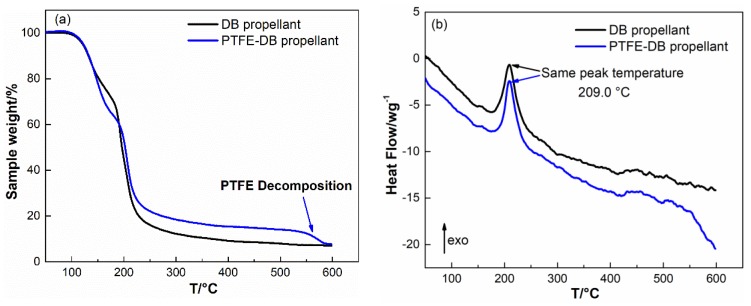
Non-isothermal TG curves (**a**) and DSC (**b**) curves of DB and PTFE-DB propellant.

The thermogravimetry analysis (TGA) of DB and PTFE-DB propellant were conducted as shown in Figure 3 to investigate the influence of PTFE on thermal behavior of DB propellant.

It is visible from Figure 3 that the DB propellant shows two stages of mass loss which correspond to the evaporation and decomposition [29] of NG (120–170 °C) and the decomposition of NC (170–230 °C), respectively. No significant difference was determined for PTFE-DB propellant in the process of NC and NG decomposition. The same temperature of decomposition peak infers that PTFE would not result in bad influence on propellant thermal stability. 

The corresponding differential scanning calorimetry (DSC) showed that there were no measurable exothermal processes for both DB and PTFE-DB propellants below 120 °C. It means that no significant decomposition of NG occurred below 120 °C [31]. Thereby, isothermal thermogravimetry analysis were carried out at temperatures below 120 °C to exclude the potential weight loss from NG decomposition.

### 4.4. Kinetics of NG Evaporation

**Figure 4:**
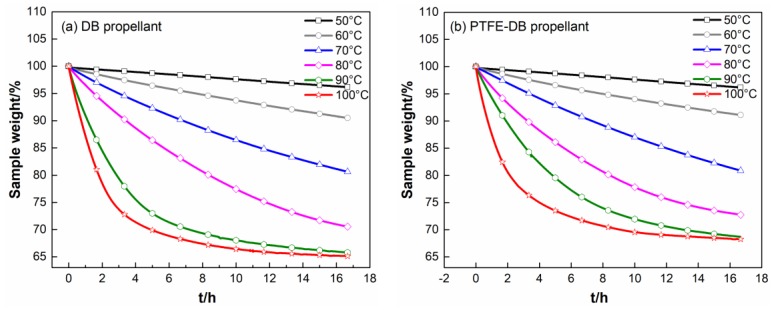
Isothermal TG curves of DB propellant (**a**) and PTFE-DB propellant (**b**) at different temperatures.

Thin plate samples with normalized size were heated at 50–100 °C with increment of 10 °C to investigate the kinetics of NG evaporation. The TGA results are shown in Figure 4. 

The obtained sample mass vs. time curve were converted to the conversion (α) vs. time according to Equation (1) [29]:

(1) $$\alpha =\frac{m_{t}-m_{0}}{m_{f}-m_{0}},$$

where m_0_ is the initial sample mass, m_f_ is the final sample mass (NG completely evaporated), and m_t_ is the instantaneous sample mass. 

**Figure 5:**
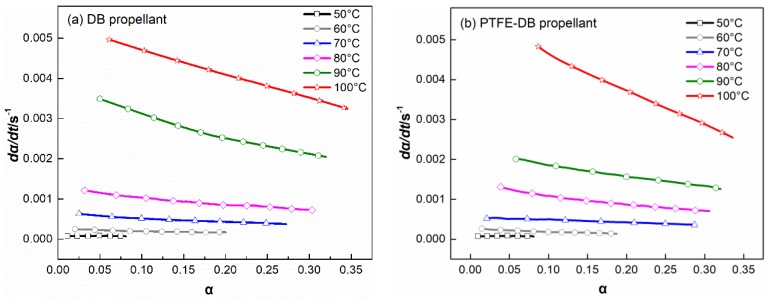
Differential conversion vs. conversion curves of DB propellant (**a**) and PTFE-DB propellant (**b**) at different temperatures.

Then the obtained α were differentiated to time and plotted vs. α as shown in Figure 5. In view of the early stage evaporation of NG in thin piece samples has been proved agree with zero order model, the curve of dα/dt vs. α could be described by the basic kinetic equation as Equation (2)

(2) $$\frac{\mathrm{da}}{\mathrm{dt}}=k_{\nu \mathrm{ap}}f\left(a\right),$$

where k_vap_ is the evaporation rate constant, and f(α) is the function which describes the dependence of the differential conversion on conversion. In the case of plasticizer evaporation from double base propellant, f(α) could be simplified as (1 − α)^n^ meaning Equation (3) [22,31,37]:

(3) $$\frac{\mathrm{da}}{\mathrm{dt}}=k_{\nu \mathrm{ap}}{\left(1-\alpha \right)}^{n},$$

It is worth noting that the exponent n is influenced by the sample size and shape, thus, the normalized sample size and shape was used in isothermal thermogravimetry analysis. To reduce the influence of variation in NG concentration on the evaporation process, the α was limited to 0.2 during fitting to Equation (3).

The values of evaporation rate constant (k_vap_) achieved by fitting to Equation (3) are shown in Table 2.

**Figure 6:**
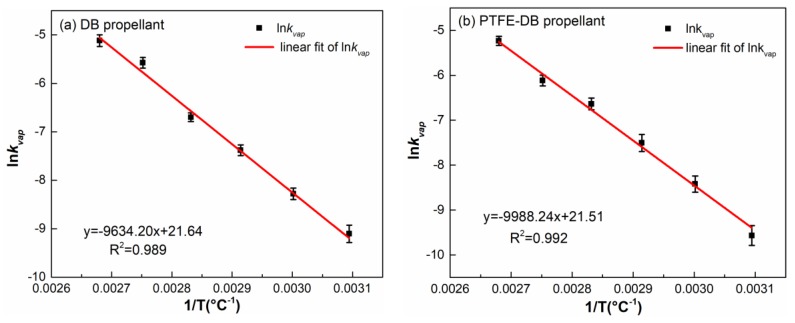
Arrhenius plots of weight loss rate of DB propellant (**a**) and PTFE-DB propellant (**b**).

From Table 2 we can see that the k_vap_ of NG in PTFE-DB propellants is slightly less than that in DB propellants under the same temperature. It means that the evaporation of NG may be partly suppressed by PTFE. To determine the influence of PTFE on evaporation of NG, Arrhenius plots (ln k_vap_ vs. l/T) with good linearity was used to calculate the active energy of evaporation. The plots of DB and PTFE-DB propellants are shown in Figure 6. The obtained kinetic parameters are shown in Table 3.

For DB propellant: E_vap_ = 80.1 kJ·mol^−1^ and A_vap_ = 2.50 × 10^9^ s^−1^, while for PTFE-DB propellant: E_vap_ = 83.0 kJ·mol^−1^ and A_vap_ = 2.19 × 10^9^ s^−1^. The determined activation energy of NG evaporation in DB propellant is close to the results of Sućeska [22]. The E_vap_ for PTFE-DB propellant slightly increased by 4% to 83.0 kJ·mol^−1^ and A_vap_ was slightly reduced by 12.4%. However, the difference between the kinetic parameters for the two propellant is very small. Thereby, the introduced PTFE may just have little suppress action in early NG evaporation of DB propellant. It is also worth noting that the surface of DB propellant is occupied by propellant components. While for PTFE-DB propellant, PTFE could reduce the percentage of propellant surface for NC and NG, which is closely related to the evaporation rate. In this regard, the reduction in evaporation may due to the decreased surface area or concentration. However, the ratio of NC/NG in PTFE-DB propellant remain the same as that in DB propellant. And the mass loss percent of PTFE-DB propellant has been converted to the total mass of NC/NG components instead of the mass of PTFE-DB propellant. If the PTFE have no influence on NG evaporation, the α for PTFE-DB propellant would be the same as that of DB propellant. Thereby, the obtained suppress effect is attributed to the action of PTFE.

### 4.5. Kinetics of NG Diffusion

As well known that the migration of NG could be well described by the Fick’s second law, i.e.,

(4) $$\frac{\partial C}{\partial t}=D\cdot \frac{\partial^{2}C}{\partial x^{2}}=D\cdot \frac{\partial }{\partial x}\cdot \left(\frac{\partial C}{\partial x}\right),$$

where C is concentration as a function of time ‘t’ and at distance ‘x’, D is diffusion coefficient. According to Cartwright [31], early stage evaporation data in a semi-infinite solid slab could also be used to calculate the diffusion coefficient due to Equation (5) derived from Equation (4) since the actual evaporation process is diffusion-limited:

(5) $$m_{t}=C_{1}+C_{2}\exp\left(-\mathrm{kt}\right),$$

where k is (𝜋^2^𝐷)/𝑙^2^, in which 𝑙 is the thick of the solid slab. Thereby, we calculated the D at a relative early stage by fitting TGA data to model Equation (5). The data used here were chose in a way similar to that of Cartwright. The achieved D were listed in Table 4.

It is visible from Table 4 that all the determined D for PTFE-DB propellant is lower than that of DB propellant. It means that PTFE limited NG diffusion as we expected. This may be mainly caused by the barrier action from the crystal PTFE molecules.

In fact, the mass loss process is related to NG evaporation and NG diffusion. The isothermal thermogravimetry analysis shown in Figure 4 has a former segment that is nonlinear with respect to time and a latter segment in which the mass of propellant decreases linearly. An analysis of these curves demonstrates that their complicated shape is due to the superposition of two processes, namely, NG evaporation and linear law NG diffusion from internal to surface at a constant rate that is zeroth order with respect to concentration. Thereby, only little fraction of TGA data could be used to fit Equation (5). In addition, Fick’s second law uses the assumption that the D is independence with NG concentration. However, D could be influenced by its concentration in practical. Thus, the calculated D with Equation (5) have some limitations. 

At former segment (Figure 4), the mass loss is associated with NG evaporation and diffusion. And the variation in NG concentration is obvious. Thus, it is complicated for the curves of isothermal thermogravimetry analysis. In the course of time, the concentration of NG in the surface layer of sample is reduced to a low level. At this time, NG diffusion from internal to propellant surface almost entirely controlled the mass loss rate. Consequently, in the latter segment of TGA curves mass of propellant decreases linearly. At this time k could be a constant related to diffusion coefficient. Usually, the Equation (2) for the α > 0.5–0.7 conditions, the following classical form Equation (6) is preferred to determine the D [38]:

(6) $$\alpha =1-\frac{8}{\pi^{2}}\exp\left(\frac{-\pi^{2}\mathrm{Dt}}{l^{2}}\right),$$

where 𝑙 is the thickness of the film. Taking the logarithm of this equation makes it possible to determine the diffusion coefficients due to Equation (7):

(7) $$\ln\left(1-\alpha \right)=\ln\left(\frac{8}{\pi^{2}}\right)-\mathrm{kt},$$

where k is (𝜋^2^𝐷)/𝑙^2^.

According this form, we calculated the diffusion rate constant and the corresponding diffusion coefficient D at different temperature. The results are listed in Table 5.

It is worth noting that the time for isothermal thermogravimetry analysis was limited to 1000 min by the instrument. The α for samples determined below 80 °C could not reach 0.6. Thus, some more tests at 110 and 120 °C were conducted to achieve more information associated with the influence of PTFE on NG diffusion. The isothermal TGA curves and corresponding plots of α vs. t for the two propellants were given in Figures S5 and S6, respectively.

It is visible from Table 5 that the order of magnitudes of diffusion coefficient of NG is 10^−13^–10^−14^ m^2^·s^−1^, agreeing with the values elsewhere [31]. The D of NG in PTFE-DB propellant is lower than that in DB propellant which is consistent with results in Table 4. This confirms that PTFE could limit NG diffusion. However, the D_1_ (calculated from early stage evaporation as shown in Table 4) is one order of magnitude larger than D_2_ (calculated from later segment TGA data as shown in Table 5) for both DB and PTFE-DB propellant. This is mainly due to the variation of NG concentration. It is well known that diffusivity is assumed independent with concentration when using Fick’s second law. While it is a function of concentration in practice. Thereby the difference between the two Ds may be mainly caused by the reduced concentration of NG, since the initial content of NG is higher than that in later segment evaporation process. From this point of view, the results of simulations may be comparable with D_1_.

In addition, the reduction of D_2_ due to PTFE is lager for the value calculated from D_1_. This is mainly caused by the inclusion of evaporation when determine the D_1_ from early stage TGA data. As mentioned above, the complicated curve shape implies the superposition of two processes. Though the diffusion of NG could be decreased by PTFE, the early stage evaporation could not be influenced obviously as shown in Table 2 and Table 3. While in the later segment of TGA data, the concentration of NG is relatively low. The mass loss rate of propellant sample in this time is almost entirely determined by NG diffusion. Thus, the calculated suppress action of PTFE due to D_1_ is weakened. The reduction of D_1_ and D_2_ imply that PTFE could suppress NG diffusion from internal to surface, which may enhance DB propellant storage performance.

### 4.6. MD Simulation

**Figure 7:**
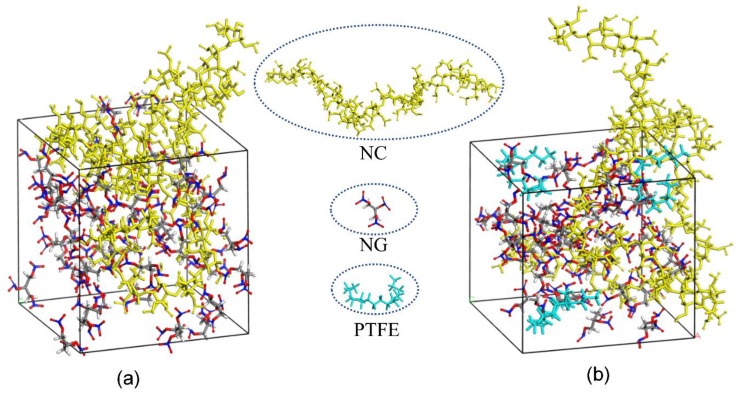
Modeling for the amorphous cells of NC/NG (**a**) and PTFE/NC/NG (**b**) in MD simulations.

Based on the MD simulation results, modeling for the amorphous cells of NC/NG and PTFE/NC/NG are shown in Figure 7. The diffusion coefficient (D) of NG can be obtained through linear regressions on the MSD vs. t curve (one-sixth the slope of the fitted regression line) [39]. The detailed results are shown in Table 6.

It can be found that the D of NG in DB propellant is a little faster than that in PTFE-DB propellant from Table 6. It indicates that the NG motion could be suppressed by PTFE in DB propellant. This may be mainly caused by the interaction between NC/NG and PTFE molecules. Since the polarity of PTFE is almost completely counteracted due to the symmetrical structure, this interaction may be mainly dispersion forces resulting from instantaneous dipoles. The simulated results infer that the interaction between PTFE and NC/NG molecules could result in an 9–21% reduction in D. 

It is worth noting that the distribution of PTFE in experimental sample is different to that in simulated sample. The fundamental purpose of simulation in this paper is to study the effect of PTFE molecules on the heat diffusion of NG. Thereby, we put PTFE in the built cell in molecule scale. While in experimental sample, there are many interfaces between nano PTFE fibers and NC/NG components. From this point of view, the reduction in D may be decreased to a large extent. However, the reduction of D from TGA data was not decreased obvious as shown in Table 4 and Table 5. Thereby, there may be some other factors limiting the migration of NG in experimental sample. Based on the fact that PTFE at the inner of the fibers cannot influence the NG molecules directly, we believe that the crystal PTFE fibers could act as inhibitions in a sense which has been proved could obvious limit NG diffusion in propellant. In summary, PTFE fibers could not only obvious improve propellant mechanical properties but also reduce the loss of NG during aging in a facile way. It is of practical importance for prolonging the service life of DB propellant and potentially expanding its application field.

## 5. Conclusions

In this work, we introduced PTFE to double base propellant and investigated the impact of PTFE on propellant mechanical properties and NG diffusion characteristics. It was found that PTFE promoted propellant tensile performances and suppressed NG diffusion. PTFE constructed a network structure in DB propellant matrix during preparation. It promoted tensile performances of DB propellant dramatically. Incorporation PTFE enhanced propellant tensile strength at 50 °C from 3.28 MPa to 5.61 MPa. The elongation at −40 °C was increased by 115% from 6.34% to 13.6%. In addition, nano PTFE fibers with high crystallinity was well dispersed in propellant matrix during propellant preparation. The NG migration in DB propellant was decreased to a large extent due to interaction between PTFE and NG molecules and the obstructing action from these fibers similar to that of inhibitions. Consequently, NG loss was largely reduced for PTFE-DB propellant during aging. The elongation was determined only 3.08% at −40 °C after aged for DB propellant while it is still high as 11.4% for PTFE-DB propellant. The significantly promoted mechanical performances and reduced diffusion of NG are of vital importance for potentially prolonging the actual service lifetime.

## Figures and Tables

**Table 1:** Tensile properties of aged double base propellants.

Sample		50 °C		20 °C		−40 °C	
		σ ^(a)^/MPa	ε ^(b)^/%	σ ^(a)^/MPa	ε ^(b)^/%	σ ^(a)^/MPa	ε ^(b)^/%
Unaged	A	3.28	33.9	11.5	19.6	51.2	6.34
	P6	5.61	69.5	15.3	51.5	64.5	13.6
1d	A	3.97	29.2	11.9	16.5	55.2	5.49
	P6	5.66	66.4	15.6	49.4	63.5	13.1
2d	A	5.44	22.5	12.9	12.3	61.6	4.41
	P6	5.84	64.2	16.2	47.7	66.1	12.4
3d	A	7.40	19.3	15.4	9.79	55.9	3.08
	P6	6.29	65.5	16.7	45.1	61.3	11.4

(a) σ: maximum tensile strength; (b) ε: elongation at maximum tensile strength.

**Table 2:** The values of evaporation rate constant (k_vap_).

Sample	k_vap_/s^−1^					
	50 °C	60 °C	70 °C	80 °C	90 °C	100 °C
DB	0.00011	0.00024	0.00059	0.00123	0.00304	0.00535
PTFE-DB	0.00007	0.00022	0.00054	0.00131	0.00221	0.00533

**Table 3:** Evaporation kinetics of NG in the two propellants.

Sample	E_vap_/(kJ·mol^−1^)	*A*_vap_
DB	80.13	2.50 × 10^9^ s^−1^
PTFE-DB	83.04	2.19 × 10^9^ s^−1^
Ref. [22]	81.9	5.6 × 10^7^ s^−1^

**Table 4:** Diffusivity of NG calculated from early stage evaporation.

Sample	D_1_/(×10^−12^ m^2^·s^−1^)							
	120 °C	110 °C	100 °C	90 °C	80 °C	70 °C	60 °C	50 °C
DB	3.38	1.84	1.35	0.783	0.285	0.151	0.0577	0.0247
PTFE-DB	3.04	1.68	1.08	0.568	0.245	0.127	0.0508	0.0217
Reduction	10.0%	8.73%	20.0%	27.4%	14.0%	15.9%	12.0%	12.2%

**Table 5:** Diffusion kinetics of NG calculated from later stage evaporation in the two propellants.

Sample	D_2_/(×10^−13^ m^2^·s^−1^)				
	120 °C	110 °C	100 °C	90 °C	80 °C
DB	5.22	4.08	3.32	2.46	1.64
PTFE-DB	3.27	2.76	2.19	1.40	1.10
Reduction	37.4%	32.2%	34.1%	43.1%	32.9%

**Table 6:** Diffusivity of NG calculated from MD simulation.

Sample	D_3_/(×10^−12^ m^2^·s^−1^)							
	120 °C	110 °C	100 °C	90 °C	80 °C	70 °C	60 °C	50 °C
DB	16.4	7.58	4.07	2.36	1.21	0.61	0.31	0.26
PTFE-DB	14.9	6.72	3.45	1.94	0.96	0.49	0.26	0.21
Reduction	9.15	11.3	15.2	17.8	20.7	19.6	16.2	19.2

## Supplementary Materials

The following are available online at http://www.mdpi.com/1996-1944/11/11/2236/s1, Figure S1. Energy profiles in dynamic simulations. (a) potential; (b) kinetics; (c) nonbond; and (d) total, Figure S2. A typical MSD vs. t curve, Table S1. Mass loss of NG in the two DB propellants. Figure S3. Stress-strain curves of the two propellants, (a) 50 °C; (b) 20 °C; (c) −40 °C. Figure S4. Crosslinking density (a) and molecular weight between crosslinking points (b). Figure S5. Isothermal TG curves of DB propellant (a) and PTFE-DB propellant (b) at different temperatures. Figure S6. Conversion vs. time curves of DB propellant (a) and PTFE-DB propellant (b) at different temperatures.
