# Supplementary material for: A Facile Way to Prolong Service Life of Double Base Propellant
Source: Materials (Basel). 2018 Nov 10;11(11):2236. doi: 10.3390/ma11112236 (PMC6266550; doi:10.3390/ma11112236)
Supplement: Supplementary file 1 [file materials-11-02236-s001.pdf]

Supplementary Materials

Article

A Facile Way to Prolong Service Life of Double Base Propellant

Shixiong Sun [1], Song Ma [1], Benbo Zhao [1], Guangpu Zhang [1] and Yunjun Luo [1,2,*]

[1] School of Materials Science and Engineering, Beijing Institute of Technology, Beijing 100081, China; sunshixiong1989@126.com (S.S.); ms2234056@163.com (S.M.); zhaobenbo@163.com (B.Z.); guangpu_0507@126.com (G.Z.)
[2] Key Laboratory for Ministry of Education of High Energy Density Materials, Beijing 100081, China;
* Correspondence: yjluo@bit.edu.cn; Tel.: +86-10-68913698; Address: School of Materials Science and Engineering, Beijing Institute of Technology, 5 South Zhongguancun Street, Beijing 100081, China.

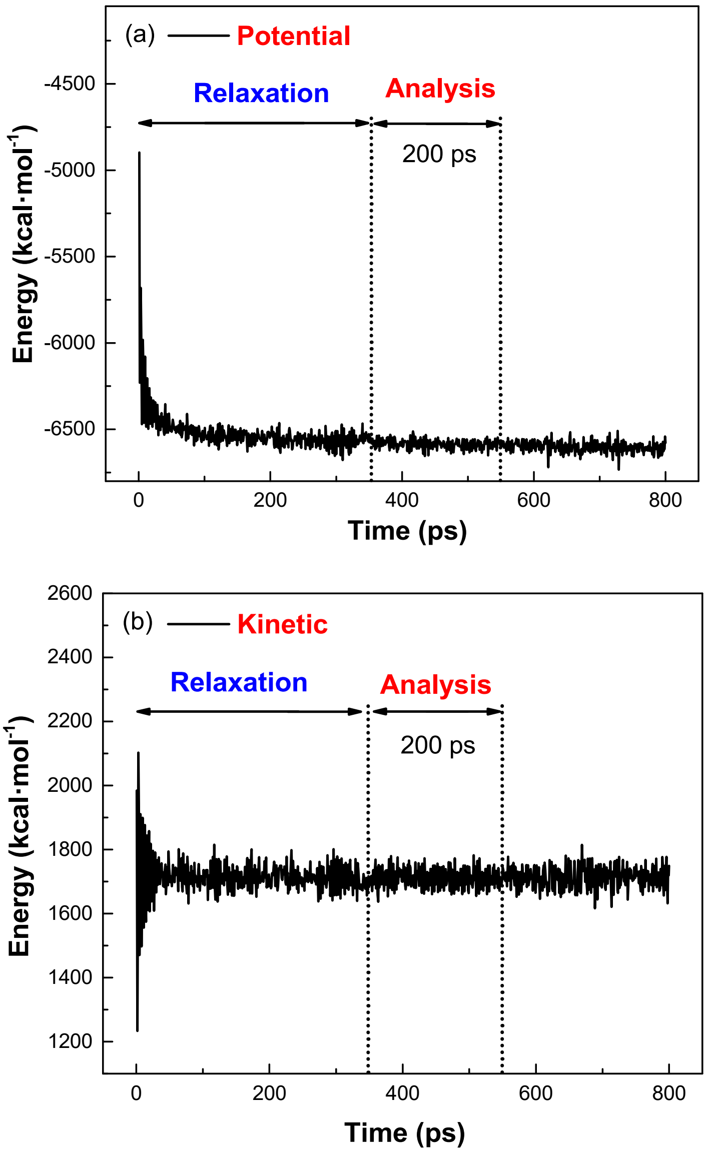

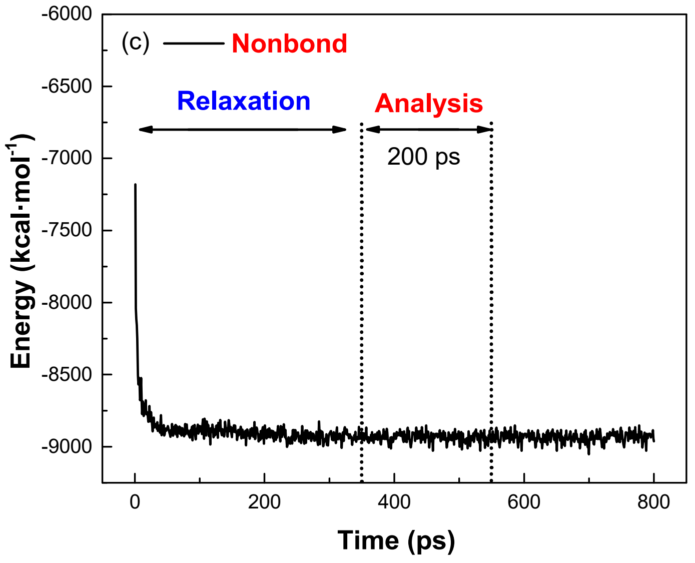

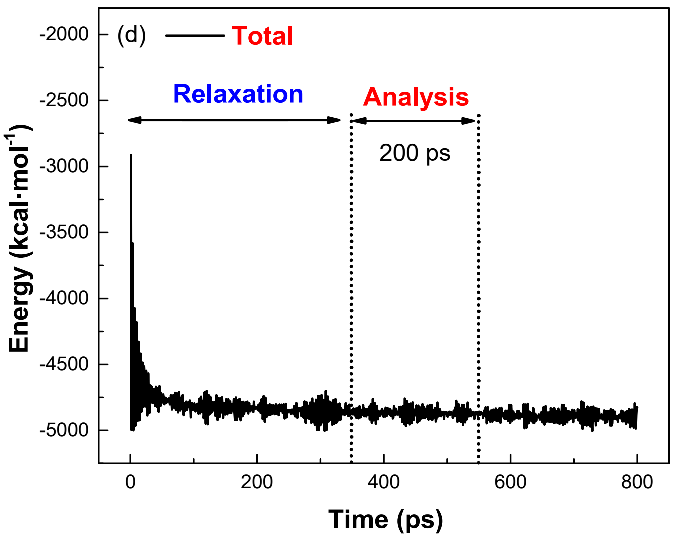

Figure S1. Energy profiles in dynamic simulations. (**a**) Potential; (**b**) kinetics; (**c**) nonbond; and (**d**) total.

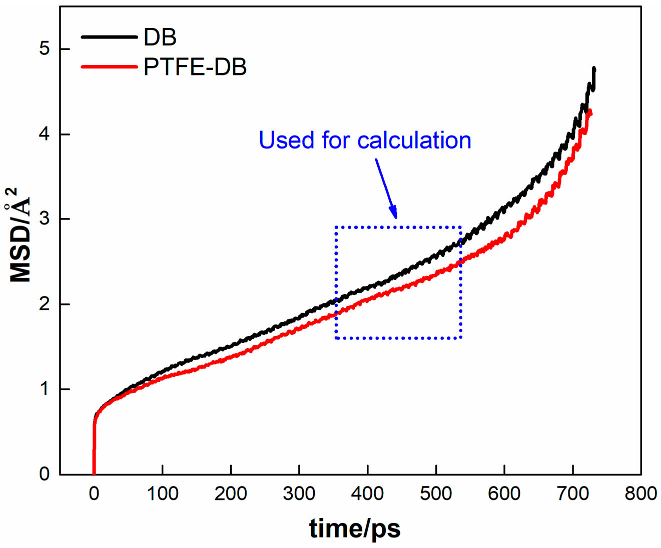

Figure S2. A typical MSD vs. t curve.

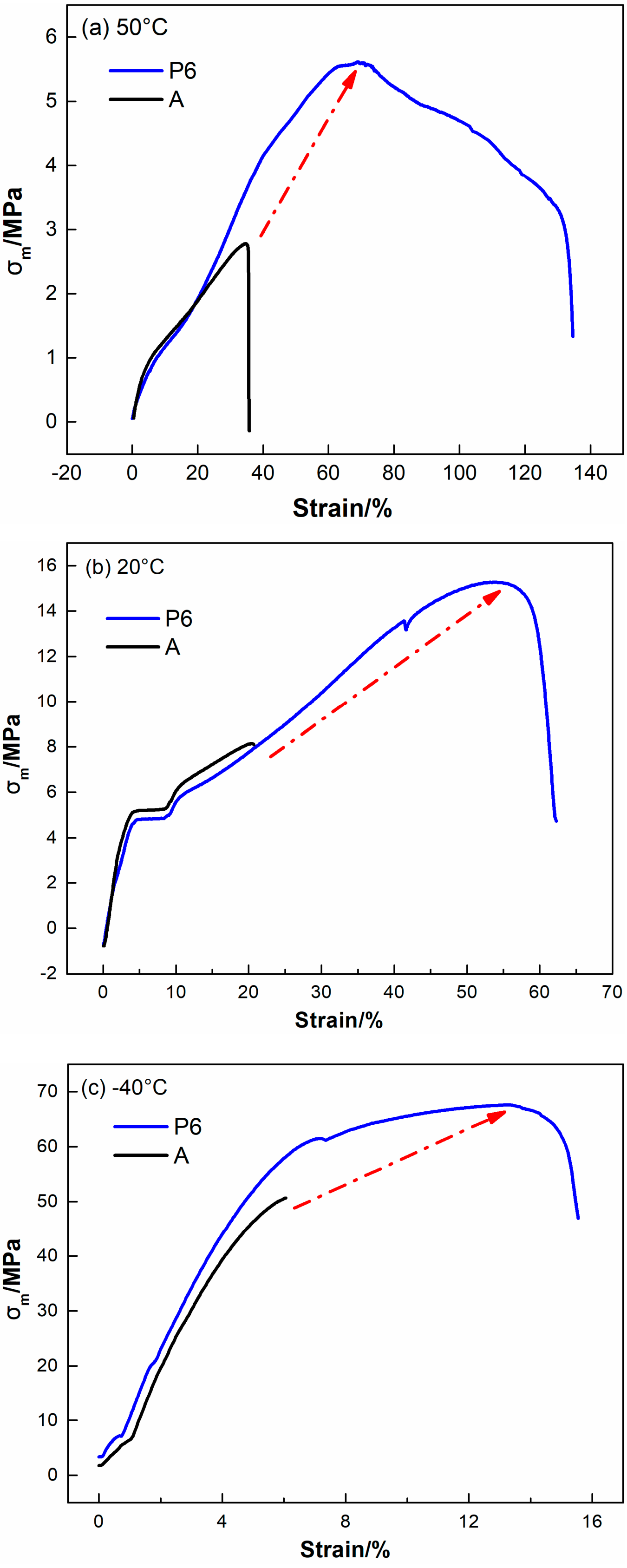

Figure S3. Stress-strain curves of the two propellants, (**a**) 50 °C; (**b**) 20 °C; and (**c**) −40 °C;

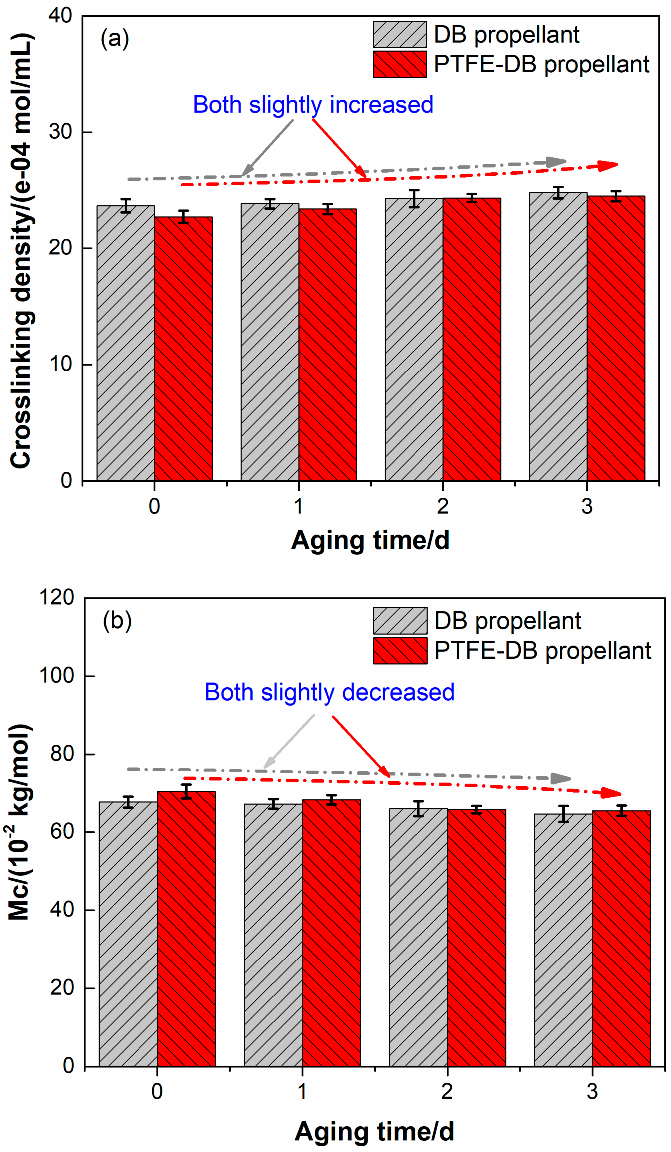

Figure S4. Crosslinking density (**a**) and molecular weight between crosslinking points (**b**).

Table S1. Mass loss of NG in the two DB propellants.

Sample	NG content/%				Reduced by/%			
	Unaged	1d	2d	3d	Unaged	1d	2d	3d
A	48.5	43.8	39.4	36.1	-	9.69	18.8	25.6
P6	48.5	45.6	43.2	40.7	-	5.98	10.9	14.0

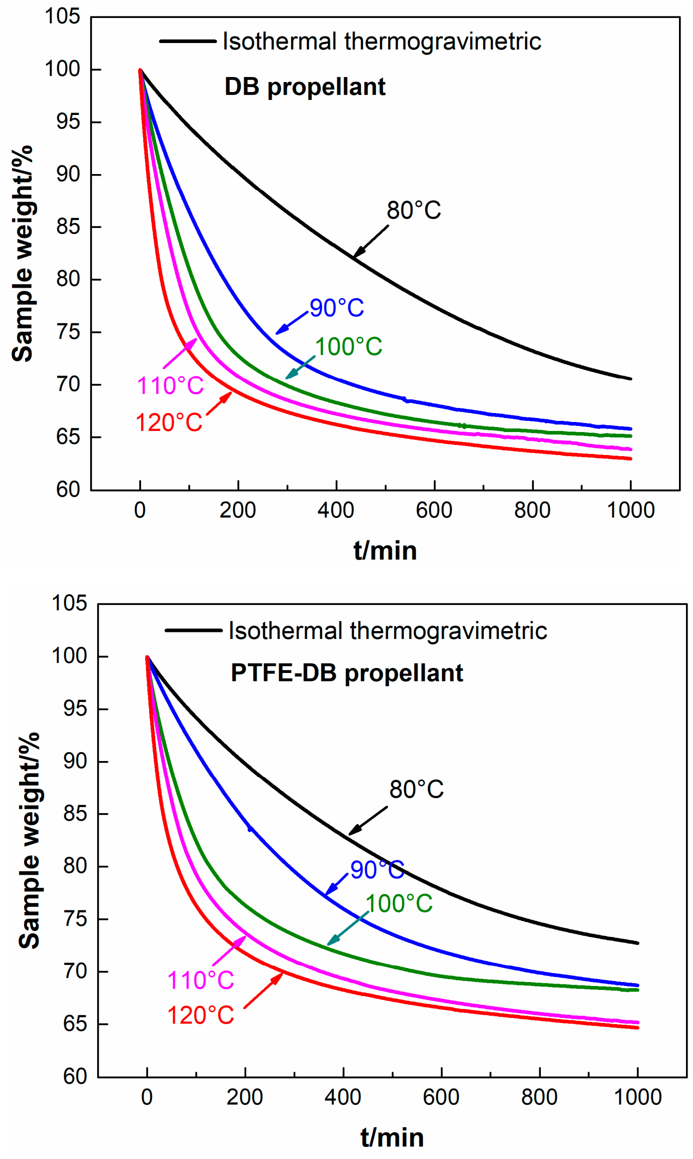

Figure S5. Isothermal TG curves of DB propellant (**a**) and PTFE-DB propellant (**b**) at different temperatures.

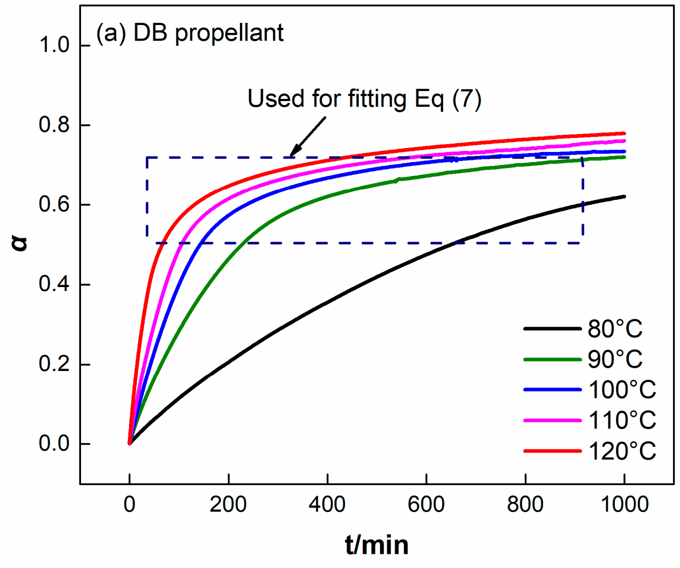

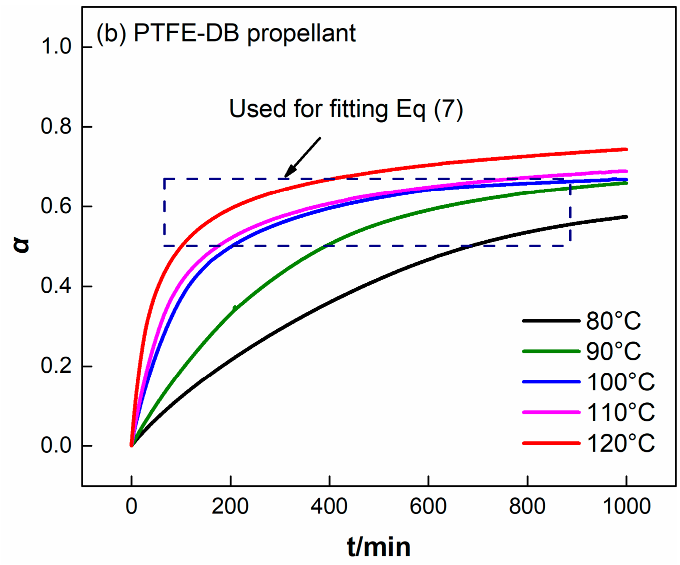

Figure S6. Conversion vs. time curves of DB propellant (**a**) and PTFE-DB propellant (**b**) at different temperatures.
